# Genome-wide analysis of hepatic LRH-1 reveals a promoter binding preference and suggests a role in regulating genes of lipid metabolism in concert with FXR

**DOI:** 10.1186/1471-2164-13-51

**Published:** 2012-02-01

**Authors:** Hansook Kim Chong, Jacob Biesinger, Young-Kyo Seo, Xiaohui Xie, Timothy F Osborne

**Affiliations:** 1 Department of Molecular Biology and Biochemistry, University of California, Irvine, CA 92697 USA; 2 Institute for Genomics and Bioinformatics, University of California, Irvine, CA 92697 USA; 3 Department of Computer Science, University of California, Irvine, CA 92697 USA; 4 Metabolic Signaling and Disease Program, Burnham Institute for Medical Research, Lake Nona FL 32827 USA; 5 Ambry Genetics, 100 Columbia #200 Aliso Viejo, California 92656 USA

**Keywords:** LRH-1, FXR, ChIP-seq, lipid metabolism

## Abstract

**Background:**

In a previous genome-wide analysis of FXR binding to hepatic chromatin, we noticed that an extra nuclear receptor (NR) half-site was co-enriched close to the FXR binding IR-1 elements and we provided limited support that the monomeric LRH-1 receptor that binds to NR half-sites might function together with FXR to activate gene expression.

**Results:**

To analyze the global pattern for LRH-1 binding and to determine whether it might associate with FXR on a whole genome-wide scale, we analyzed LRH-1 binding to the entire hepatic genome using a non-biased genome-wide ChIP-seq approach. We identified over 10,600 LRH-1 binding sites in hepatic chromatin and over 20% were located within 2 kb of the 5' end of a known mouse gene. Additionally, the results demonstrate that a significant fraction of the genome sites occupied by LRH-1 are located close to FXR binding sites revealed in our earlier study. A Gene ontology analysis revealed that genes preferentially enriched in the LRH-1/FXR overlapping gene set are related to lipid metabolism. These results demonstrate that LRH-1 recruits FXR to lipid metabolic genes. A significant fraction of FXR binding peaks also contain a nuclear receptor half-site that does not bind LRH-1 suggesting that additional monomeric nuclear receptors such as RORs and NR4As family members may also target FXR to other pathway selective genes related to other areas of metabolism such as glucose metabolism where FXR has also been shown to play an important role.

**Conclusion:**

These results document an important role for LRH-1 in hepatic metabolism through acting predominantly at proximal promoter sites and working in concert with additional nuclear receptors that bind to neighboring sites

## Background

Nuclear receptors are signal-regulated transcription factors that control a wide range of biological processes and influence many human diseases [1]. Nuclear receptor activity is controlled by the binding of natural small molecules or ligands including hormones and metabolites and many synthetic compounds have been designed to mimic these natural regulators [2]. The ability of nuclear receptors to alternate between activation and repression in response to specific ligands is mediated by differential binding of non-DNA binding co-regulators, including co-activators and co-repressors [3]. In general, this switch is mediated through a conformational change in the ligand binding pocket of the nuclear receptor leading to dissociation of co-repressors and interaction with co-activators.

In addition to the non-DNA binding ligand-gated co-regulators, nuclear receptor activity can also be influenced by the binding of other DNA binding partner proteins that can interact with the nuclear receptors to form a cis-regulatory module to enhance or repress the transcription of select target genes [3].

The liver receptor homolog-1 (LRH-1; NR5A2)) is expressed mainly in the liver, intestine, exocrine pancreas, and ovary [4-6] and plays a role in the regulation of bile acid, cholesterol, and steroid hormone homeostasis. It belongs to a nuclear receptor subfamily that includes steroidogenic factor 1 (SF-1; NR5A1). LRH-1 was cloned independently by several groups and it received many names, including pancreas homolog receptor 1 (PHR-1), fetoprotein transcription factor (FTF), CYP7A1 promoter binding factor (CPF), human B1 binding factor (hB1F) [7].

Unlike nuclear receptors that form heterodimers with RXR to bind to their response element, LRH-1 regulates target genes by binding as a monomer to DNA response elements with consensus sequence 5'PyCAAGGPyCPu3' [7], which is similar to a "half-site" recognized by dimeric receptors. LRH-1 is involved in the regulation of genes, which participate in steroid, bile acid and cholesterol homeostasis [8]. Recent structural studies for LRH-1 and SF-1 revealed a phospholipid located in the binding pocket of the protein crystal suggesting phospholipids might function as natural ligands [9,10]. Whereas the physiological relevance of the interaction between LRH-1 and putative phospholipid ligands remains to be fully appreciated, a recent study supports the role for specific phospholipids as regulatory agonists for LRH-1 in vivo [11].

LRH-1 also has a key role early in development where it activates expression of Oct4, which is required to maintain pluripotency at the earliest stages of both embryonic development and in ES cell differentiation [12]. In fact, a recent study showed that LRH-1 could replace Oct4 in the re-programming of mouse somatic cells into pluripotent cells by presumably activating Oct4 [13].

In our analysis of FXR binding to hepatic chromatin, we showed that LRH-1 could function as a partner transcription factor for FXR on a small set of target genes through binding to a nuclear receptor half-site that was co-enriched with the FXR IR-1 element on a genome-wide scale [14]. To determine how global the association between FXR and LRH-1 might be and to analyze LRH-1 more broadly, the binding of LRH-1 to the whole liver genome was accomplished by a non-biased genome wide ChIP-seq analysis in liver using an LRH-1 antibody to enrich LRH-1 target regions that were subsequently sequenced using Applied Biosystems' SOLiD (Sequencing by Oligonucleotide Ligation and Detection) System. The studies demonstrate that LRH-1 binds to over 10,6000 sites in the genome with a significant fraction located close to FXR binding sites identified in our earlier study. Gene ontology grouping revealed that the genes preferentially bound by both FXR and LRH-1 are involved in lipid metabolism suggesting that LRH-1 targets FXR for activation of genes of lipid metabolism. These data also suggest that additional monomeric nuclear receptors such as RORs and NR4As may also bind to NR half-sites close to FXR elements that are not occupied by LRH-1, which could target FXR to different gene clusters involved in other key areas of metabolism.

## Results and Discussion

### Identification of the Hepatic Cistrome for LRH-1

In our previous studies of genome-wide binding for FXR, our analysis revealed that an additional nuclear receptor (NR) half-site was present in 71% of the FXR/RXR binding IR-1 sites from our liver FXR ChIP-seq dataset [14]. We also demonstrated that the IR-1 and additional NR half-sites were located relatively close together with most occurrences containing the two motifs within 50 bases of each other [14]. This finding suggested that FXR regulates gene expression in combination with a co-binding monomeric nuclear receptor.

LRH-1 is a prominent monomeric liver NR that binds to half-site elements and we showed that a few of the FXR target promoters also bound LRH-1 [14]. To both analyze the genome-wide binding for LRH-1 and to determine whether it might be associated with FXR binding on a genome-wide scale, we performed a ChIP-seq analysis with hepatic chromatin after enrichment with an LRH-1 antibody. Chromatin prepared from livers of six C57BL6 mice was pooled and processed for ChIP with an antibody to LRH-1 or a control IgG as described in Methods. The quality of the chromatin and specificity of the LRH-1 antibody were confirmed by comparative site-specific ChIP analysis using known FXR binding sites in the promoters of SHP, Pemt, Pcx, and Abca4 (Additional File 1). Chromatin enriched by the LRH-1 antibody produced a significantly increased qPCR signal for LRH-1 binding to these promoters relative to chromatin pulled down with a control IgG fraction (Additional File 1).

Next, DNA from the LRH-1 antibody enriched chromatin was subjected to ChIP-seq using the Applied Biosystems' SOLiD platform. The sequencing libraries were prepared according to the standard SOLiD System 2.0 Fragment Library Preparation protocol and the quality of ChIPed DNA, including DNA fragmentation and library amplification, was evaluated by using Agilent BioAnalyzer before running the sequencing reactions. Most DNA fragments were between 200-600 bp in size for both samples (Additional File 2). The DNA fragments between ~200-300 bp were selected for library preparation and SOLiD sequencing.

**Figure 1:**
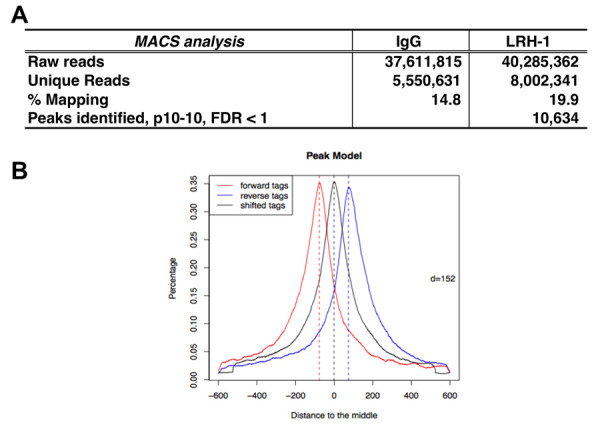
**MACS analysis for LRH-1 ChIP-seq**. (A) Summary of ChIP-seq analysis for LRH-1 binding to DNA in hepatic chromatin by MACS. Given *mfold *32 and sonication size (*bw*) 300 bp, MACS searched 2*bw *window area across the genome to find genomic peaks with tags more than *mfold *enriched relative to a random tag genome distribution. The results were obtained using the parameters of p-value cutoff 1 × 10^-10 ^and false discovery rate (FDR) 1%. (B) Peak model built by MACS. MACS estimated the *d *for LRH-1 ChIP-seq data.

The data generated more than 40 million independent sequencing reads (Table 1). The individual 39 bp reads were filtered for high quality, as well as for alignment and unique placement in the mouse reference genome by using SOLiD™ BioScope™ Software (Life Technologies). This resulted in 8.3 million uniquely mapped reads corresponding for the IgG and 10.6 million for the LRH-1 enriched sample (Table 1). However, we applied an even more stringent cutoff mapping quality scores (MAPQ > 5) and obtained ~5.5 million for IgG and ~8 million reads for LRH-1 enriched samples which were used for further analysis (Table 1 and Figure 1A).

**Table 1:** Summary of SOLiD ChIP-seq analysis

Uniquely Mapped Reads					
**Antibody**	**Total** **Raw Reads**	**MAPQ > 1**	**% Mapping**	**MAPQ > 5**	**% Mapping**

IgG	37,611,815	**8,343,715**		**5.550,631**	**14.8**
LRH-1	40,285,362	**10,635,029**		**8,002,341**	**19.9**

The reads were generated from one quadrant of SOLiD ChIP-seq system. All reads were filtered for high quality reads, as well as for alignment and unique placement in the mouse reference genome by using the SOLiD BioScope Software. Antibody and total raw reads (black). Analyzed uniquely mapped reads used for analysis (Bold)

To identify LRH-1 binding peaks, we used Model-based Analysis of ChIP-seq (MACS), which was designed to analyze data generated by short read sequencers such as from the SOLiD platform [15] to first estimate peak size and location, using BED files as an input. The distance between the modes of the forward and reverse peaks in the alignments, defined as '*d*', was 152 bp for the LRH-1 ChIP-seq data (Figure 1B). Using stringent p-value and false discovery rate (FDR) cutoffs of ≤ 1 × 10^-10 ^and ≤ 1% respectively, we identified 10,634 genomic sites occupied by LRH-1 protein (Figure 1A).

**Figure 2:**
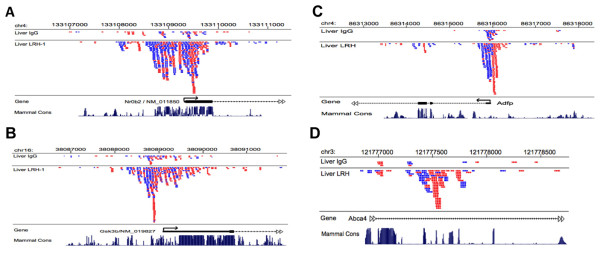
**Representative view of a LRH-1 ChIP-seq peak**. The novel LRH-1 binding sites, mapped onto University of California at Santa Cruz (UCSC) genome browser, were identified in several genes presented here. Shown are chromosomal locations according to the July 2007 Mouse Genome Assembly (mm9). Blue and red tags represent sequence reads from opposite DNA strands showing approximately equal distribution as expected. (A) Nr0b2 (SHP). (B) Adfp (adipose differentiation related protein). (C) Gsk3b (Glycogen Synthase Kinases-3b). (D) Abca4 (ABC transporter 4).

**Figure 3:**
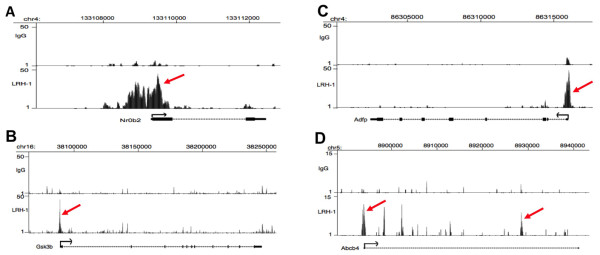
**Representative view of putative LRH-1 peaks and the entire locus of respective genes using bedGraph format**. The novel FXR binding sites are mapped onto University of California at Santa Cruz (UCSC) genome browser. Shown are chromosomal locations of each peak and its gene according to the July 2007 Mouse Genome Assembly (mm9). (A) Nr0b2 (SHP). (B) Adfp (adipose differentiation related protein). (C) Gsk3b (Glycogen Synthase Kinases-3b). (D) Abca4 (ABC transporter 4).

The aligned sequence reads were displayed as a track onto the mouse reference genome using the University of California at Santa Cruz (UCSC) genome browser (http://genome.ucsc.edu/index.html), and visual inspection of several sites confirmed that the LRH-1 peaks identified by MACS correspond to sites of over-represented sequence tags. For the examples shown in Figure 2, sequence reads corresponding to different DNA strands are colored in blue and red respectively for the SHP, Adfp, Gsk3b and Abca4 gene associated binding peaks. The peaks for SHP, Adfp or Gsk3b were distributed in the promoter regions, whereas that for Abca4 was located in an intron. We also inspected LRH-1 binding peaks by using the bedGraph format that allows a display of continuous-valued ChIP-seq data in track format using the UCSC genome browser. This showed LRH-1 binding peaks and extended regions from the entire locus of the respective genes (Figure 3).

### Mapping of LRH-1 binding peaks

**Figure 4:**
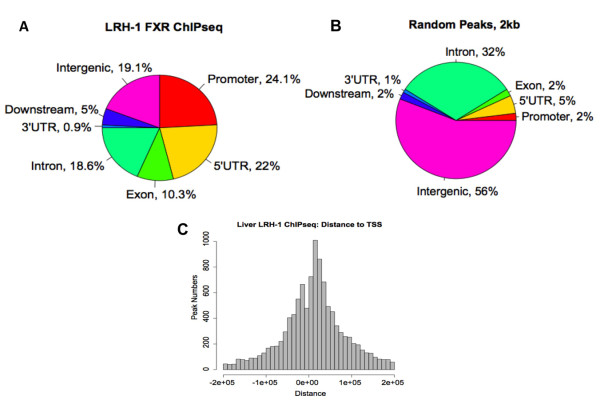
**Mapping of LRH-1 binding regions**. (A) Mapping of LRH-1 binding peaks on genome-wide scale relative to RefSeq mouse genes. (B) Mapping for random peaks. The 'promoter' and 'downstream' are defined as 2 KB of 5' or 3' flanking regions. Intergenic region refers to all locations other than 'promoter', '5' UTR', 'exon', 'intron', '3'UTR', or 'downstream' (C) Distance from the summit of each LRH-1 peak to the TSS of the nearest RefSeq gene. An arbitrarily located site of the same length in each peak showed a non-enriched distribution pattern as reported previously [27].

When we evaluated where the LRH-1 binding peaks were located with respect to mRNA encoding genes, we were surprised to find that LRH-1 binding sites were predominantly located in the promoter regions (2 kb 5', 24.1%), and 5'UTR (22%) relative to the transcription start site (TSS) for known genes (Figure 4A). Altogether, this accounts for 46% of the total LRH-1 binding events, suggesting a strong preference for TSS proximal binding by LRH-1. In contrast, when the genomic location for randomly generated peaks of similar size was estimated, the random peaks were predominantly localized within intergenic (56%) and intron (32%) regions, with only 2% positioned within 2 kb of a TSS (Figure 4B). Thus, the 24.1% for LRH-1 binding sites to within 2 KB of a TSS is a highly non-random occurrence. Next, we examined the distance from the summit of each LRH-1 peak to the TSS of the nearest identified gene. The distribution shown in Figure 4C provides a visual demonstration that LRH-1 binding peaks were enriched close to TSS for known genes.

### Motif analysis for LRH-1 binding by MEME

**Figure 5:**
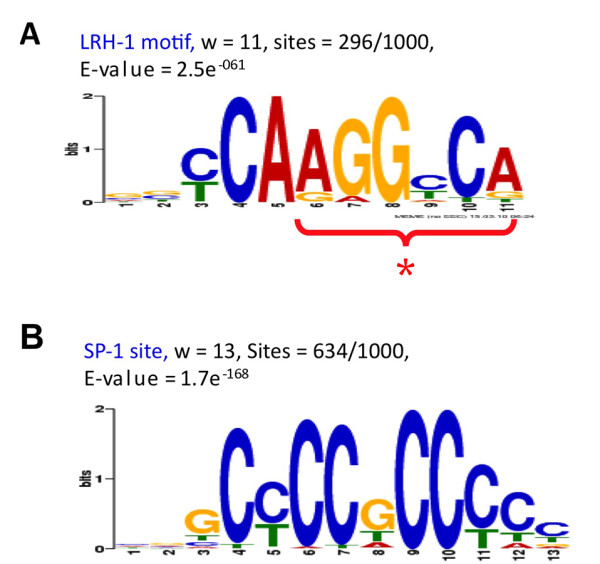
**Motif Analysis of LRH-1 peaks by MEME program**. Consensus LRH-1-binding motif Weblogo found within the top 1000 peaks identified by LRH-1 ChIP-seq using MEME program. (A) Our LRH-1 motif identified by MEME. (B) SP-1 site, identified by MEME. * indicates a nuclear receptor half-site

The motif finding program MEME [16] was used to search for enriched motifs in the peaks from our LRH-1 ChIP-seq data set. We found two motifs that were represented with a very high score. One corresponds to a NR half site of 5'-CCAAGGTCA-3' (MOTIF 2; sites = 296/1000; E-value = 2.5e^-061^) (Figure 5A). 30% (296/1000) of all input peaks contained at least one of these half-site elements. This indicates that our genome wide analysis of *in vivo *binding sites is consistent with previous studies on the half-site for binding of LRH-1 (5'-CAGGGTCA-3') '[17]. Additionally, this result is consistent with the genome-wide binding analyses fore an epitope-tagged and over-expressed LRH-1 in cultured embryonic stem cells reported previously [13]. The other top-scoring motif identified by the MEME program was the GC box corresponding to a site for Sp1 binding (E-value = 1.7e^-168^), (Figure 5B). Sp1 is a transcription factor that is ubiquitously expressed and contains three C_2_H_2_-type zinc fingers as DNA binding domain [18]. The Sp1 site was enriched at both promoter proximal and distal LRH-1 sites. There were no other transcription factor motifs that were significantly enriched in our analyses

**Figure 6:**
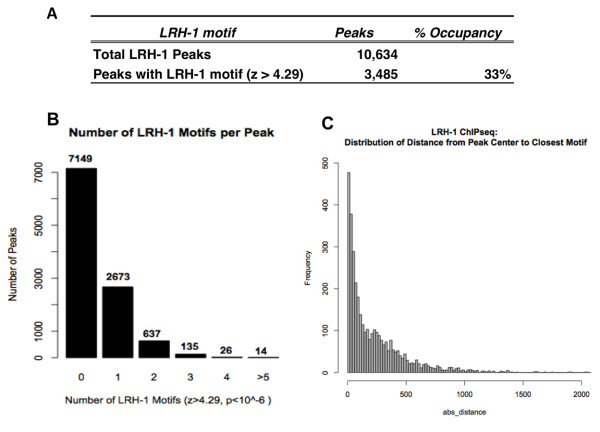
**Motif analysis for LRH-1 binding peaks**. (A) Summary of LRH-1 motif analysis. (B) Number of LRH-1 motif in a peak identified by SOLiD ChIP-seq (z > 4.29). (C) Distribution of the distance from the best LRH-1 motif to the summit of each peak with a LRH-1 site. An arbitrarily located site of the same length in each peak showed a non-enriched distribution pattern as reported previously [27].

A position weight matrix (PWM) for the LRH-1 motif from the MEME analysis was calculated and used to scan all of the LRH-1 peaks again using a more stringent z-score cutoff of 4.29 (p < 10^-6^) for motif identification. Using this stringent criterion, a half-site LRH-1 motif was present in 33% (3485/10634, z-score > 4.29) of the LRH-1 peaks from the MACS analysis (Figure 6A). Among the peaks containing the LRH-1 motif, most contain one motif element but there are some peak regions that contain more than one (Figure 6B).

Next, we calculated the distance from the best LRH-1 site in each LRH-1 motif-containing peak to the corresponding peak summit. Theoretically, this is the most likely location of the actual site of LRH-1-DNA interaction. By this analysis, the NR half-site elements were preferentially located at the peak-summits relative to randomly placed motifs of a similar size. This observation is consistent with the theoretical prediction that the ChIP-seq peak mapping technique with small sequence reads accurately identifies the actual site of protein-DNA recognition and provides more confidence that the motif containing the half-site is actually the site of recognition for LRH-1 (Figure 6C).

### Co-occupancy by peaks for LRH-1 and FXR

**Figure 7:**
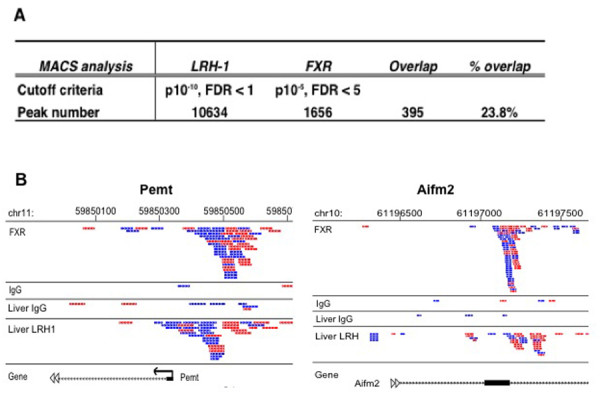
**Analysis of co-occupancy of LRH-1 ChIP-seq peak with FXR binding sites identified by MACS**. (A) Comparison of ChIP-seq analysis for LRH-1 binding in hepatic chromatin with FXR binding peaks. (B) The LRH-1 binding sites for Pemt and Aifm2, mapped onto UCSC genome browser, were inspected for co-occupancy by FXR. Blue and red tags represent sequence reads from opposite DNA strands. Left panel, Pemt (phosphatidylethanolamine N-methyltransferase); Right panel, Aifm2 (apoptosis-inducing factor 2, mitochondrion).

To investigate whether LRH-1 binding sites were enriched close to the sites of FXR binding from our previous study, we compared the ChIP-seq dataset for LRH-1 binding sites with our previous dataset for FXR binding peaks. This analysis showed that 23.8% of all FXR binding peaks were located close to LRH-1 peaks (Figure 7A). We also visually inspected the locations of several of the LRH-1 binding sites with respect to neighboring FXR binding peaks, using peak distribution tracks in the UCSC genome browser. This comparison for LRH-1 binding sites at the Pemt and Aifm2 loci is shown in Figure 7B and clearly shows the close apposition of the binding peaks for the two different ChIP-seq data sets.

### Genes located close to the LRH-1 binding sites in liver

There were 395 overlapping peaks between LRH-1 and FXR binding (Figure 7A) that are located within 10 KB of 367 RefSeq genes. We used the DAVID Gene Ontology (GO) PANTHER 'Biological Process' term (http://david.abcc.ncifcrf.gov/) [19] to provide information on the genes that were co-occupied by LRH-1 and FXR. This analysis showed that there was a strong enrichment for genes in lipid metabolic processes, steroid and cholesterol metabolism (Table 2). The most significantly enriched genes were associated with 'cellular lipid metabolic process' (FDR = 0.0002%) and many of the genes in this category are predicted to regulate cholesterol homeostasis (Sec14l2, Scarb1, Srebp2, Lcat, Fdft1, Prkag2 and Ldlrap1).

**Table 2:** Summary of DAVID Gene Ontology analysis of genes near LRH-1 binding regions

Category	Term	GO Term	Count	%	P value	Benjamini	FDR
GOTERM_BP	GO:0044255	**Cellular lipid metabolic process**	25	9.73	1.07E-06	0.00552	0.002
GOTERM_BP	GO:0006629	**Lipid metabolic process**	26	10.12	3.88E-06	0.01003	0.0074
GOTERM_BP	GO:0008152	metabolic process	141	54.86	1.82E-05	0.031	0.0348
GOTERM_BP	GO:0008202	**Steroid metabolic process**	10	3.89	2.95E-04	0.31791	0.5618
GOTERM_BP	GO:0044237	Cellular metabolic process	125	48.64	3.00E-04	0.26791	0.5722
GOTERM_BP	GO:0008203	**Cholesterol Metabolic Processes**	7	2.72	3.97E-04	0.29075	0.7557
GOTERM_BP	GO:0016125	**Sterol metabolic process**	7	2.72	6.63E-04	0.3885	1.259
GOTERM_BP	GO:0044238	Primary metabolic process	123	47.86	8.15E-04	0.41083	1.5454
GOTERM_BP	GO:0008610	**Lipid biosynthetic process**	12	4.67	8.48E-04	0.38705	1.608
GOTERM_BP	GO:0009058	Biosynthetic process	33	12.84	0.001103	0.43623	2.0869
GOTERM_BP	GO:0044248	Cellular catabolic process	16	6.23	0.002877	0.74332	5.356
GOTERM_BP	GO:0032787	Monocarboxylic acid metabolic process	10	3.89	0.004361	0.84904	8.0104
GOTERM_BP	GO:0006066	Alcohol metabolic process	11	4.28	0.005747	0.89995	10.428
GOTERM_BP	GO:0006631	**Fatty acid metabolic process**	8	3.11	0.008909	0.86381	15.716
GOTERM_BP	GO:0019752	Carboxylic acid metabolic process	15	5.84	0.010269	0.97193	17.899
GOTERM_BP	GO:0006082	Organic acid metabolic process	15	5.84	0.010587	0.96837	18.4

367 genes close to LRH-1 peaks that overlap with FXR peaks were used to group into enriched functionally important categories using the PANTHER "Biological Process" term

### Correlation between LRH-1 binding and FXR dependent gene regulation

**Figure 8:**
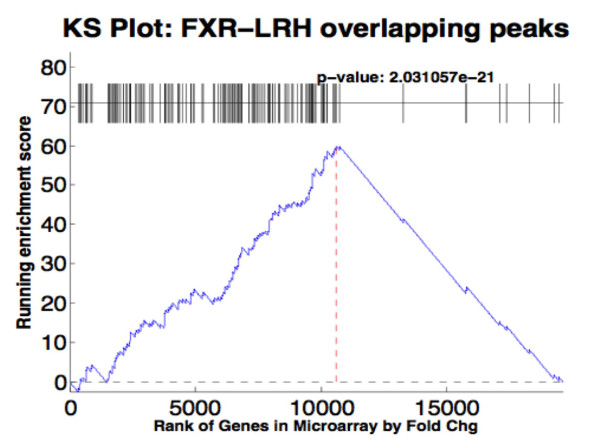
**Peak validation using Kolmogorov-Smirnov (KS) plot**. The gene list for the LRH-1 ChIP-seq peaks that overlap with FXR ChIP-seq peaks was compared for their correlation to a set of genes that were activated by infection of primary mouse hepatocytes with a recombinant adenovirus expressing the constitutive FXRα2-VP16 hybrid protein as described in the text. Genes in the expression microarray were ranked by absolute fold change (A) or fold change (B) (x-axis) and the graph plots the running enrichment score.

We reasoned that if the co-occurrence of FXR and LRH-1 binding sites was functionally important then the genes associated with LRH-1 sites should be statistically correlated with a functional data set for FXR dependent gene expression. Thus, we analyzed the gene list from the MACS analysis for LRH-1 binding peaks for overlap with genes that were preferentially activated by an FXR expressing adenovirus [14] using a gene set enrichment analysis (GSEA) function and the modified Kolmogorov-Smirnov (KS) test [20]. This KS plot distributes results from a gene expression microarray rank ordered for fold change on the X-axis and the occurrence of a gene from the ChIP-seq data set is then scanned for going from high to low fold change. The presence or absence of a ChIP-seq identified gene is scored on the Y-axis with a running enrichment score. This analysis showed a highly significant running enrichment score because the genes identified by LRH-1 ChIP-seq that overlap with FXR binding peaks were preferentially located toward the top of the differentially expressed gene list ranked by fold change in gene expression (Figure 8, p = 1.06e^-07^). Thus, it is highly likely that LRH-1 is a global co-regulator for FXR dependent gene expression.

In a previous report, we identified a nuclear receptor half-site that was co-enriched with FXR binding IR-1 sites in liver chromatin [14]. LRH-1 is a liver enriched monomeric nuclear receptor that binds to half-site elements, so we hypothesized that LRH-1 would be a good candidate for binding the adjacent half-site to function as a FXR co-regulatory protein in liver chromatin. In fact, we presented a limited amount of evidence for this on a handful of FXR target genes [14], but it was important to extend this association to a genome-wide scale. To accomplish this goal, a genome-wide SOLiD ChIP-seq analysis was performed using chromatin enriched with an LRH-1 antibody. The SOLiD ChIP-seq data for LRH-1 binding generated more than 40 millions reads of 39 bp sequence tags. The ultra-high throughput SOLiD DNA sequencing platform is able to produce more than 400 million tags of 35-50 bp per run, and the high read numbers contribute to high sensitivity and signal-to-noise ratios, and to relative comprehensiveness for the genome. 10,634 genomic LRH-1 binding sites were identified with a high degree of confidence (p-value ≤ 1 × 10^-10^, FDR ≤ 1%) (Table 1 and Figure 1).

When we used the motif finding program MEME [16] to search for enriched motifs in the peaks from our LRH-1 ChIP-seq dataset, we found a motif (5'-CCAAGGTCA-3') containing a nuclear receptor half-site (MOTIF 2) (Figure 5) and 33% of all input peaks contained at least one LRH-1 motif (Figure 6). Our genome wide analysis of *in vivo *binding sites is also consistent with our previous studies for the half-site preference for binding of LRH-1 on the Fasn promoter (5'-CAGGGTCA-3') '[17].

On a genome-wide scale, the LRH-1 binding sites were localized mainly in proximal promoters (24%) and 5'UTR (22%) regions, whereas similar to other nuclear receptors analyzed to date, FXR binding occurs primarily in distal intergenic regions (44%) and introns (32%), with only 10% localizing to proximal promoter [14].

The ChIP-seq analysis demonstrated that LRH-1 binding sites are located close to ~24% of the FXR-binding sites (Figure 7). This represents a highly significant degree of co-localization with a p < 10 ^-6 ^that was calculated by sampling a control set of peaks with the same size distribution. The FXR/LRH-1 co-association was highly significant for both promoter proximal and non-proximal binding sites. This provides strong support for our hypothesis that LRH-1 is a key hepatic co-regulatory transcription factor for FXR.

We also analyzed the association of genes located close to FXR and LRH-1 binding sites relative to genes activated by FXR using a gene set enrichment analysis. The LRH-1 associated genes were localized within a set of FXR activated genes that were rank-ordered for differential expression after infection of primary hepatocytes with a control or a constitutively active FXR-VP16 fusion protein ([14], Figure 8). The corresponding Kolmogorov-Smirnov (KS) plot showed there was a high degree of correlation of the two data sets providing additional evidence that LRH-1 regulates genes in conjunction with FXR.

Because 76% of the LRH-1 binding sites were not located close to FXR elements, these results also predict that LRH-1 regulates gene expression without FXR as well. Consistent with this hypothesis LRH-1 has been shown to play a key role in regulating gene expression along with LXR as well [17,21,22].

The gene ontology analysis in Table 2 indicated that the genes co-regulated by FXR and LRH-1 are associated with lipid metabolic processes. It is likely that other nuclear receptors, such as RORs, NR4a's, ERR's and Reverb, that also bind as monomers to an isolated NR half-site, may target FXR to genes involved in other physiological responses. In fact, the NR4a nuclear receptors are involved in physiological processes including glucose metabolism and DNA repair [23] and these two GO categories were ranked just behind lipid metabolism as the most significantly associated pathways for FXR binding in our previous study [14]. When we analyzed a list of NR4a responsive genes from microarray studies summarized in a previous report [23], we noticed that 14/48 of these target genes were found in our FXR target gene list. This is a highly significant correlation (p = 8.8 e^-8^), which provides strong support for this model of FXR pathway targeting.

Another relevant monomeric nuclear receptor where data from mouse liver is available is for the Reverb-α transcriptional regulator [24]. In fact, recent studies suggest it is a repressor of lipogenic gene expression during the light phase of the diurnal cycle [24]. When the overlap for genome-wide binding of Reverb-α at ZT 10 (the light phase) and LRH-1 in our study was evaluated, we found that there was a highly significant overlap (18% of LRH-1 peaks at p < 10^-6^) which is consistent with Reverb-α inhibiting lipogenesis during the light phase of the diurnal cycle at least partly through inhibiting genes that are activated by LRH-1 [24].

## Conclusions

Our studies contribute to understanding the mechanism by which FXR and LRH-1 cooperatively regulate lipid metabolic process and suggest a generalized model for how FXR may be targeted to additional metabolic processes such as glucose and bile acid metabolism through association with distinct half-site binding monomeric nuclear receptors. The details and molecular mechanism of this cooperation remain to be elucidated. However, it is possible that the ability of FXR to function along with LRH-1 and other co-factors such as chromatin remodeling complexes at the adjacent sites results in synergistic effects on transcription activation. Future studies are necessary to characterize the chromatin context in which FXR and LRH-1 binding occurs, including histone modification profiles such as methylation or acetylation, binding site accessibility, as well as recruitment of other cofactors, by using rapidly advancing genome-wide binding approaches.

## Methods

### Chromatin immunoprecipitation sequencing (ChIP-seq) using the SOLiD platform

Six-week-old C57BL6 male mice were fed a standard chow diet [25]. All animals were sacrificed at the end of the dark cycle and ChIP assays from liver were performed as previously described [14,25]. The liver chromatin from all six animals were pooled for analysis. Chromatin was extracted and subjected to an immunoselection process, which required the use of antibodies against LRH-1 (PP-H2325-00; R&D Systems) or mouse IgG (Sigma) as a control. To prepare samples for the SOLiD ChIP-seq, after isolating the ChIP-enriched DNA, gene-specific enrichment for some known FXR target genes including SHP, Pemt, Pcx, and Abca4 in the LRH-1 chromatin relative to IgG control chromatin was verified. Approximately 20 ng of ChIP enriched DNA or control DNA was processed by the Sanford-Burnham Medical Research Institute Genomics Core Facility (Orlando, FL) for high throughput DNA sequencing using SOLiD system. The libraries for the samples were prepared according to the standard SOLiD System 2.0 Fragment Library Preparation protocol. Then templated bead generation for each library was performed according to SOLiD System 2.0 Users Guide standard protocols. Each sample was deposited on a quadrant of the slide at a target bead density of 60-70 k beads/panel.

### Quantitative PCR, microarray analysis

Manual ChIP confirmation on the randomly selected putative FXR target genes from lipid metabolism category was achieved by quantitative PCR (qPCR) method [26]. Final ChIPed and control DNA samples were analyzed in triplicate with L32 as internal control. For this assay, we used pre-designed and validated qPCR primer specific to the peak regions containing LRH-DNA interaction and an additional co-regulatory site, and measured genomic DNA promoter region sequence enrichment within ChIPed samples.

### ChIP-seq data analysis

#### Preprocessing sequence data

The ultra high read tag numbers of the SOLiD system contributes to high sensitivity, relative comprehensiveness for the mouse genome, and enables very robust statistical power required to map and accurately characterized the protein-DNA interactions of an entire genome. Like other sequencing technologies, it measures fluorescence intensities from dye-labeled molecules to determine the sequence of DNA fragments. The location of the sequence reads from SOLiD System and their frequency, which measures the degree of enrichment over the control, was revealed using currently available SOLiD sequencing analytical tools including SAMtools (http://samtools.sourceforge.net/).

The SOLiD ChIP-seq dataset was analyzed to determine peaks which contain binding sites of LRH-1 to its target genes. Short reads of 39-bp were produced from Applied Biosystem's (ABI) SOLiD (Sequencing by Oligonucleotide Ligation and Detection) System, and mapped to a reference genome by Life Technologies using SOLiD™ BioScope™ Software, allowing two mismatch. Short sequence reads that mapped to simple and complex repeats or that were not unique by chance were removed from the analysis. The resulting mapped file was in SAM ("Sequence Alignment/Map") format, and we converted the SAM files to BED files using SAMTools (http://samtools.sourceforge.net/), which can provide various utilities for manipulating alignments in the SAM format, including sorting, indexing, merging and generating alignments in a per-position format. The BED files which contain chromosomal start and stop positions were used as input to downstream processing, as well as visualization in the UCSC Genome Browser (http://genome.ucsc.edu/index.html).

#### Finding peaks using MACS

To determine where the LRH-1 bound to the genome, we looked for areas where there were significantly more enriched reads mapped in the ChIP sample than in the IgG. This was accomplished using MACS [15] with the parameters of *mfold *32, *bandwidth *300 bp, p-value 1 × 10^-10^, and FDR 1%.

#### Distance to LRH-1 sites from the summit of each peak

MACS provides a summit for every peak, which can be regarded as the center of the peak. It is where there is the maximum number of overlapping reads, and is the most likely location of the binding site. For each peak with an LRH-1 site, we determined the distance from the best LRH-1 site to this summit. If they overlapped, we score the distance as zero. To give a sense of the enrichment, we evaluated an arbitrarily located site of the same length in each peak, determined the distance to the summit, and plotted the results on the same histogram.

#### Distance from peak to TSSs

For each LRH-1 peak, the distance from the peak to the nearest transcription start site was determined, and plotted. The transcription start sites (TSSs) were taken from a RefSeq file obtained from NCBI. The background was determined by placing peaks at random locations on the genome and by determining distances to TSSs.

#### Motif analysis

DNA sequences for LRH-1 binding regions were retrieved using Galaxy (http://main.g2.bx.psu.edu) and used for motif search using MEME [16]. MEME represents motifs as position-dependent letter-probability matrices (PWM). The PWM was used to find a score for the top-scoring LRH-1 sequence; each letter in the sequence has a likelihood given in the PWM, these were summed to find a score for the sequence, with a higher score meaning it is more likely to be the motif in question. We used the PWM to find scores for every position along an entire chromosome (excepting coding and repeat regions), and found the average score and standard deviation. Then when a new sequence was tested, we obtained its score from the PWM, subtracted the average, and divided by the standard deviation. This provided us a z-score for any sequence, which was converted into a p-value via a standard normal curve.

The position weight matrix (PWM) for the LRH-1 motif from the MEME analysis was used to scan all our LRH-1 peaks again using a more stringent z-score cutoff of 4.29 (p < 10^-6^).

#### Annotation of genes and gene ontology (GO) analysis

All LRH-1 binding sites were assigned to nearest genes based on the Mus musculus NCBI m37 genome assembly (mm9; July 2007). GO analysis of LRH-1 target genes was conducted by using the NIH Database for Annotation, Visualization, and Integrated Discovery (DAVID; http://david.abcc.ncifcrf.gov/) [19]. This analysis was used to classify the nearest gene list into functionally related gene groups by using 'PANTHER Biological Process' term.

#### Kolmogorov-Smirnov analysis

The obtained LRH-1 ChIP-seq data was compared with an expression microarray data set for FXR dependence [14] by using a Kolmogorov-Smirnov (KS) plot, a modified method of gene set enrichment analysis (GSEA) [20]. The KS plot tests the null hypothesis that the ranks of the genes identified by ChIP-seq is uniformly distributed throughout the FXR expression microarray. A KS plot was obtained by calculating the running sum statistics for our ChIP-seq gene set to observe enrichment in the ranked gene list from expression microarray data.

## List of Abbreviations used

LRH: liver receptor homologue; FXR: farnesoid × receptor: ChIP: chromatin immunoprecipitation; GO: gene ontology; KS: Kolmogorov-Smirnov; TSS: transcription start site

## Competing interests statement

The authors declare that they have no competing interests.

## Authors' contributions

HKC, JB and YKS performed experiments, HKC, JB, XX and TO analyzed data, HKC and TO wrote the manuscript. All authors have read and approve of the final manuscript

## Supplementary Material

Additional file 1 **LRH-1 ChIP of selected LRH target gnes**. This file contains a qPCR analysis of 4 separate promoters after ChIP analysis for liver chromatin. This is essential to show the specificity of the LRH-1 antibody Click here for file

Additional file 2 **ChIP-seq library quality control**. an aliquot of the ChIP-seqlibrary was analyzed for fragment size using the Agilent Bioanalyzer. Click here for file

## References

[B1] Lonard DM Lanz RB O'Malley BW Nuclear receptor coregulators and human disease Endocr Rev 2007 28 5 575 587 10.1210/er.2007-0012 17609497

[B2] Chen T Nuclear receptor drug discovery Curr Opin Chem Biol 2008 12 4 418 426 10.1016/j.cbpa.2008.07.001 18662801

[B3] Glass CK Rosenfeld MG The coregulator exchange in transcriptional functions of nuclear recpetors Genes & Dev 2000 14 121 141 10652267

[B4] Falender AE Lanz R Malenfant D Belanger L Richards JS Differential expression of steroidogenic factor-1 and FTF/LRH-1 in the rodent ovary Endocrinology 2003 144 8 3598 3610 10.1210/en.2002-0137 12865342

[B5] Pare JF Malenfant D Courtemanche C Jacob-Wagner M Roy S Allard D Belanger L The fetoprotein transcription factor (FTF) gene is essential to embryogenesis and cholesterol homeostasis and is regulated by a DR4 element The Journal of biological chemistry 2004 279 20 21206 21216 10.1074/jbc.M401523200 15014077

[B6] Schoonjans K Dubuquoy L Mebis J Fayard E Wendling O Haby C Geboes K Auwerx J Liver receptor homolog 1 contributes to intestinal tumor formation through effects on cell cycle and inflammation Proceedings of the National Academy of Sciences of the USA 2005 102 6 2058 2062 10.1073/pnas.0409756102 15684064 PMC548586

[B7] Fayard E Schoonjans K Annicotte JS Auwerx J Liver receptor homolog 1 controls the expression of carboxyl ester lipase The Journal of biological chemistry 2003 278 37 35725 35731 10.1074/jbc.M302370200 12853459

[B8] Lee YK Moore DD Liver receptor homolog-1, an emerging metabolic modulator Front Biosci 2008 13 5950 5958 18508634 10.2741/3128

[B9] Krylova IN Sablin EP Moore J Xu RX Waitt GM MacKay JA Juzumiene D Bynum JM Madauss K Montana V Lebedeva L Suzawa M Williams JD Williams SP Guy RK Thornton JW Fletterick RJ Willson TM Ingraham HA Structural analyses reveal phosphatidyl inositols as ligands for the NR5 orphan receptors SF-1 and LRH-1 Cell 2005 120 3 343 355 10.1016/j.cell.2005.01.024 15707893

[B10] Ortlund EA Lee Y Solomon IH Hager JM Safi R Choi Y Guan Z Tripathy A Raetz CR McDonnell DP Moore DD Redinbo MR Modulation of human nuclear receptor LRH-1 activity by phospholipids and SHP Nature structural & molecular biology 2005 12 4 357 363 10.1038/nsmb910 15723037

[B11] Lee JM Lee YK Mamrosh JL Busby SA Griffin PR Pathak MC Ortlund EA Moore DD A nuclear-receptor-dependent phosphatidylcholine pathway with antidiabetic effects Nature 2011 474 7352 506 510 10.1038/nature10111 21614002 PMC3150801

[B12] Gu P Goodwin B Chung AC Xu X Wheeler DA Price RR Galardi C Peng L Latour AM Koller BH Gossen J Kliewer SA Cooney AJ Orphan nuclear receptor LRH-1 is required to maintain Oct4 expression at the epiblast stage of embryonic development Molecular and cellular biology 2005 25 9 3492 3505 10.1128/MCB.25.9.3492-3505.2005 15831456 PMC1084298

[B13] Heng JC Feng B Han J Jiang J Kraus P Ng JH Orlov YL Huss M Yang L Lufkin T Lim B Ng HH The nuclear receptor Nr5a2 can replace Oct4 in the reprogramming of murine somatic cells to pluripotent cells Cell Stem Cell 2010 6 2 167 174 10.1016/j.stem.2009.12.009 20096661

[B14] Chong HK Infante AM Seo YK Jeon TI Zhang Y Edwards PA Xie X Osborne TF Genome-wide interrogation of hepatic FXR reveals an asymmetric IR-1 motif and synergy with LRH-1 Nucleic Acids Res 2010 38 18 6007 6017 10.1093/nar/gkq397 20483916 PMC2952856

[B15] Zhang Y Liu T Meyer CA Eeckhoute J Johnson DS Bernstein BE Nussbaum C Myers RM Brown M Li W Liu XS Model-based analysis of ChIP-Seq (MACS) Genome Biol 2008 9 9 R137 10.1186/gb-2008-9-9-r137 18798982 PMC2592715

[B16] Bailey TL Discovering novel sequence motifs with MEME Curr Protoc Bioinformatics 2002 Chapter 2 Unit 2 4 10.1002/0471250953.bi0204s00 18792935

[B17] Matsukuma KE Wang L Bennett MK Osborne TF A key role for orphan nuclear receptor liver receptor homologue-1 in activation of fatty acid synthase promoter by liver × receptor The Journal of biological chemistry 2007 282 28 20164 20171 10.1074/jbc.M702895200 17522048

[B18] Wierstra I Sp1: emerging roles--beyond constitutive activation of TATA-less housekeeping genes Biochemical and biophysical research communications 2008 372 1 1 13 10.1016/j.bbrc.2008.03.074 18364237

[B19] Dennis G Jr Sherman BT Hosack DA Yang J Gao W Lane HC Lempicki RA DAVID: Database for Annotation, Visualization, and Integrated Discovery Genome Biol 2003 4 5 P3 10.1186/gb-2003-4-5-p3 12734009 PMC193660

[B20] Subramanian A Tamayo P Mootha VK Mukherjee S Ebert BL Gillette MA Paulovich A Pomeroy SL Golub TR Lander ES Mesirov JP Gene set enrichment analysis: a knowledge-based approach for interpreting genome-wide expression profiles Proceedings of the National Academy of Sciences of the USA 2005 102 43 15545 15550 10.1073/pnas.0506580102 16199517 PMC1239896

[B21] Lu TT Makishima M Repa JJ Schoonjans K Kerr TA Auwerx J Mangelsdorf DJ Molecular basis for feedback regulation of bile acid synthesis by nuclear receptors Molecular cell 2000 6 3 507 515 10.1016/S1097-2765(00)00050-2 11030331

[B22] Goodwin B Jones SA Price RR Watson MA McKee DD Moore LB Galardi C Wilson JG Lewis MC Roth ME Maloney PR Willson TM Kliewer SA A regulatory cascade of the nuclear receptors FXR, SHP-1, and LRH-1 represses bile acid biosynthesis Molecular cell 2000 6 3 517 526 10.1016/S1097-2765(00)00051-4 11030332

[B23] Pearen MA Muscat GE Minireview: Nuclear hormone receptor 4A signaling: implications for metabolic disease Molecular endocrinology (Baltimore, Md) 2010 24 10 1891 1903 10.1210/me.2010-0015 PMC5417389 20392876

[B24] Feng D Liu T Sun Z Bugge A Mullican SE Alenghat T Liu XS Lazar MA A circadian rhythm orchestrated by histone deacetylase 3 controls hepatic lipid metabolism Science (New York, NY) 2011 331 11 March 2011 1315 1319 10.1126/science.1198125 PMC3389392 21393543

[B25] Bennett MK Seo Y-K Datta S Shin D-J Osborne TF Selective Binding of SREBP isoforms and Co-Regulatory Proteins to Promoters for Lipid Metabolic Genes in Liver The Journal of biological chemistry 2008 283 15628 15637 10.1074/jbc.M800391200 18413311 PMC2414284

[B26] Seo YK Chong HK Infante AM Im SS Xie X Osborne TF Genome-wide analysis of SREBP-1 binding in mouse liver chromatin reveals a preference for promoter proximal binding to a new motif Proceedings of the National Academy of Sciences of the USA 2009 106 33 13765 13769 10.1073/pnas.0904246106 19666523 PMC2728968

[B27] Seo YK Jeon TI Chong HK Biesinger J Xie X Osborne TF Genome-wide Localization of SREBP-2 in Hepatic Chromatin Predicts a Role in Autophagy Cell metabolism 2011 13 4 367 375 10.1016/j.cmet.2011.03.005 21459322 PMC3086264

