# Supplementary material for: Genome-wide analysis of hepatic LRH-1 reveals a promoter binding preference and suggests a role in regulating genes of lipid metabolism in concert with FXR
Source: BMC Genomics. 2012 Feb 1;13:51. doi: 10.1186/1471-2164-13-51 (PMC3295688; doi:10.1186/1471-2164-13-51)
Supplement: Additional file 1 — LRH-1 ChIP of selected LRH target gnes. This file contains a qPCR analysis of 4 separate promoters after ChIP analysis for liver chromatin. This is essential to show the specificity of the LRH-1 antibody [file 1471-2164-13-51-S1.PDF]

SFigure S1

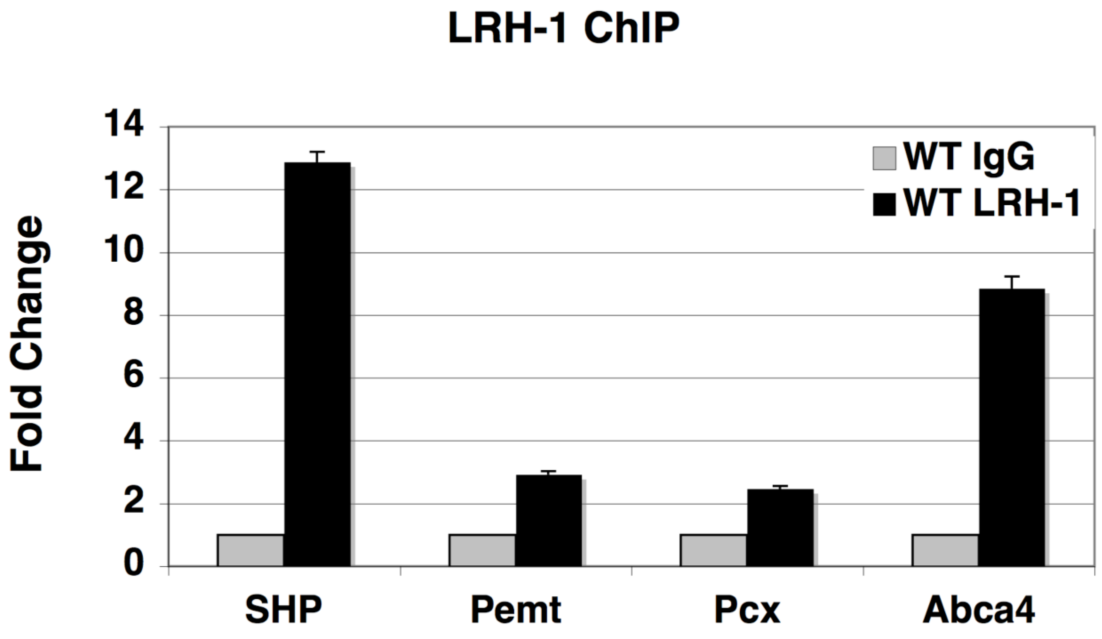

Confirmation of Chromatin and Antibody by qPCR. Binding of LRH-1 to promoters of 4 known LRH-1 target genes (Chong HK, Infante AM, Seo YK, Jeon TI, Zhang Y, Edwards PA, Xie X, Osborne TF: **Genome-wide interrogation of hepatic FXR reveals an asymmetric IR-1 motif and synergy with LRH-1**. *Nucleic Acids Res* 2010, **38**(18):6007-6017) in liver chromatin was quantified after precipitation of chromatin with an antibody to LRH-1. Chromatin from WT mouse liver was precipitated with either a control antibody fraction (IgG) or an LRH-1 antibody (LRH-1) as indicated.
