# Supplementary material for: Genome-wide analysis of hepatic LRH-1 reveals a promoter binding preference and suggests a role in regulating genes of lipid metabolism in concert with FXR
Source: BMC Genomics. 2012 Feb 1;13:51. doi: 10.1186/1471-2164-13-51 (PMC3295688; doi:10.1186/1471-2164-13-51)
Supplement: Additional file 2 — ChIP-seq library quality control. an aliquot of the ChIP-seqlibrary was analyzed for fragment size using the Agilent Bioanalyzer. [file 1471-2164-13-51-S2.PDF]

Figure S2

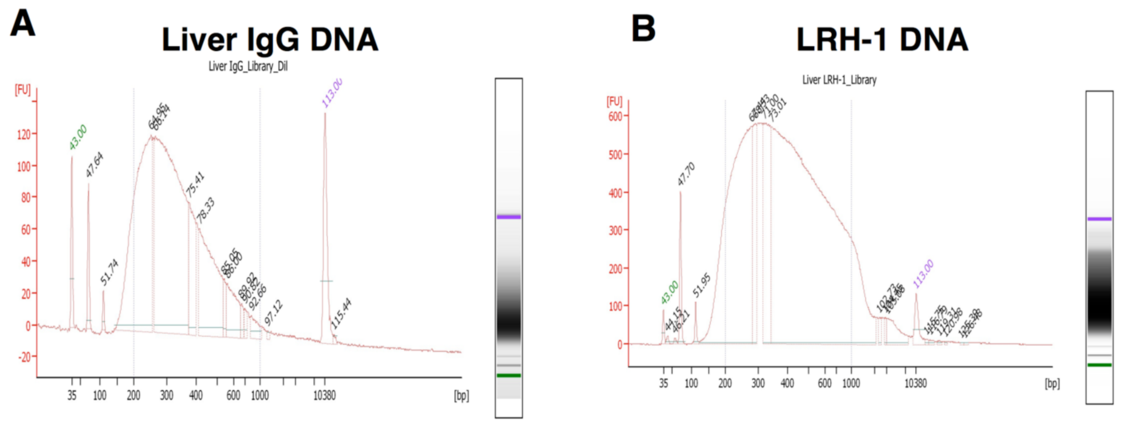

ChIP-seq library quality control. After preparation of the ChIP-seq library, a fraction of the library was loaded onto the Agilent Bioanalyzer and afterwards, the size and distribution was visualized as shown.
